# Polarized ^3^*He* Spin Filters for Slow Neutron Physics

**DOI:** 10.6028/jres.110.043

**Published:** 2005-06-01

**Authors:** T. R. Gentile, W. C. Chen, G. L. Jones, E. Babcock, T. G. Walker

**Affiliations:** National Institute of Standards and Technology, Gaithersburg, MD 20899-0001; Hamilton College, Clinton, NY 13323; University of Wisconsin at Madison, Madison, Wisconsin

**Keywords:** ^3^He, helium, neutron, optical pumping, polarization, spin-exchange, spin filter

## Abstract

Polarized ^3^He spin filters are needed for a variety of experiments with slow neutrons. Their demonstrated utility for highly accurate determination of neutron polarization are critical to the next generation of betadecay correlation coefficient measurements. In addition, they are broadband devices that can polarize large area and high divergence neutron beams with little gamma-ray background, and allow for an additional spin-flip for systematic tests. These attributes are relevant to all neutron sources, but are particularly well-matched to time of flight analysis at spallation sources. There are several issues in the practical use of ^3^He spin filters for slow neutron physics. Besides the essential goal of maximizing the ^3^He polarization, we also seek to decrease the constraints on cell lifetimes and magnetic field homogeneity. In addition, cells with highly uniform gas thickness are required to produce the spatially uniform neutron polarization needed for beta-decay correlation coefficient experiments. We are currently employing spin-exchange (SE) and metastability-exchange (ME) optical pumping to polarize ^3^He, but will focus on SE. We will discuss the recent demonstration of 75 % ^3^He polarization, temperature-dependent relaxation mechanism of unknown origin, cell development, spectrally narrowed lasers, and hybrid spin-exchange optical pumping.

## 1. Introduction

Polarized ^3^He spin filters are needed for a variety of experiments with slow neutrons. Their demonstrated utility for highly accurate determination of neutron polarization [1, 2] are critical to the next generation of beta-decay correlation coefficient measurements. [3, 4] In addition, they are broadband devices that can polarize large area and high divergence neutron beams with little gamma-ray background, and allow for an additional spin-flip for systematic tests. These attributes are relevant to all neutron sources, but are particularly well-matched to time of flight analysis at spallation sources. For these reasons, ^3^He spin filters are an integral part of the ongoing effort to measure the parity violating gamma ray asymmetry in the absorption of neutrons by hydrogen (“npdγ”) [5].

As presented at this conference, [6, 7] the current approach for accurate neutron polarimetry at the Institut Laue-Langevin is to use crossed supermirrors to polarize the neutron beam and an “opaque” spin filter to analyze the beam. In this case, the demand on the ^3^He spin filters are low because neither high polarization nor a highly uniform thickness are required. In contrast, the approach planned for spallation source experiments is to polarize the beam with a ^3^He spin filter, in which case the achievable statistical accuracy is strongly dependent on the figure of merit of the polarizer. For experiments that require accurate polarimetry, a highly uniform neutron polarization is needed. The focus of this paper is on the status and future of the technology for ^3^He spin filters with these properties. Additional practical issues for spin filters include decreased constraints on cell lifetimes and magnetic field homogeneity.

Polarized ^3^He spin filters are based on the spin dependence of the ^3^He-neutron capture cross section. For the ^3^He spin parallel to the neutron spin, the thermal capture cross section is essentially zero, whereas for the spins anti-parallel, the cross section is 10 666 b at a neutron velocity v = 2200 m/s. [8] Because of the simple 1/*υ* ^3^He-neutron absorption cross section, a ^3^He spin filter can serve well as a broadband neutron polarizer, which makes it particularly relevant to spallation neutron sources. The similarities between the formulae for transmission and polarization allow the polarization of an initially unpolarized, monochromatic, neutron beam to be determined by measurements of transmission alone. [9, 10] At a spallation source, fitting the transmission vs wavelength allows extraction of the neutron polarization. In addition, the neutron polarization can also be determined by directly fitting a beta-decay asymmetry that is proportional to the neutron polarization. [3] Approaches to accurate polarimetry at a reactor source have been proposed [11, 12].

Achieving and maintaining the highest ^3^He polarization in a neutron spin filter can have a significant impact on the feasibility of a long term experiment in slow neutron physics. The time required to reach a given statistical uncertainty in the measurement of a small asymmetry that is proportional to the neutron polarization is inversely proportional to a figure of merit $P_{n}^{2}T_{n}$, where *P*_n_ is the neutron polarization and *T*_n_ is the neutron transmission. *P*_n_ = tanh (*n****σ****lP*_He_) and *T*_n_ = *T*_0_ cosh (*n****σ****lP*_He_), where *n* is the density of ^3^He atoms in the cell, ***σ*** is the wavelength-dependent neutron absorption cross section, *l* is the length of the cell, *P*_He_is the ^3^He polarization and *T*_0_ = *T*_e_ exp (−*n****σ****l*) is the transmission through the unpolarized cell (T_e_ is the transmission through the empty cell). Assuming a spin filter with a thickness chosen to maximize this figure of merit (*n****σ****l* ≈ 1.8), a ^3^He polarization of 75 % yields *P*_n_ = 0.88 and *T*_n_ = 0.29 (assuming a typical value of 0.87 for *T*_e_). A decrease in ^3^He polarization from 75 % to 60 % will increase a one year experiment by a half a year, i.e., the figure of merit is roughly proportional to the square of the ^3^He polarization.

We are currently employing spin-exchange optical pumping (SEOP) [13, 14] and metastability-exchange (MEOP) [15] optical pumping to polarize ^3^He, but will focus on SEOP because it is best suited for the continuous, long term operation typical of fundamental physics experiments with slow neutrons. We present developments and issues in the science and technology of SEOP for spin filter applications, including temperature-dependent relaxation (Sec. 2.1), cell development (Sec. 2.2), spectrally narrowed diode lasers (Sec. 2.3), and hybrid SEOP (Sec. 2.4). We currently obtain 75 % ^3^He polarization in cells up to 0.5 L in volume. Whereas obtaining this level of polarization in even larger cells is simply a matter of increasing the laser power, a hybrid SEOP approach may be a more versatile alternative. However, improving the polarization will require understanding and eliminating a temperature-dependent relaxation of unknown origin.

## 2. Developments in Spin-Exchange Optical Pumping

In the SEOP method, electronic polarization is produced by optical pumping of alkali atoms (usually rubidium), and the polarization is transferred to the ^3^He nuclei by the hyperfine interaction during collisions. The ^3^He polarization *P*_He_ is given by

$$P_{\text{He}}=P_{\text{Rb}}\frac{k_{\text{se}}\left[\text{Rb}\right]}{k_{\text{se}}\left[\text{Rb}\right]\left(1+X\right)+\Gamma_{r}}$$ (1)

where *P*_Rb_ is the rubidium polarization, *k*_se_ is the spin-exchange rate constant, [Rb] is the Rb density, and *Γ*_r_ is the room temperature relaxation rate of the ^3^He gas in the cell containing the gas [16]. *X* accounts for the recent observation [17] that the ^3^He relaxation rate increases linearly with the Rb density with a slope that exceeds the spin-exchange rate constant. Until recently, it was assumed that aside from a small contribution from anisotropic spin-exchange, [18] *X* would be zero. We recently reported a typical value for *X* of 0.33, [16] which limits the maximum ^3^He polarization to 75 %. In practice, the Rb density is varied by varying the temperature of the cell. The origin of this temperature-dependent relaxation is unknown, and further studies are discussed in Sec. 2.1.

Because the cross section for spin-exchange collisions is small, the polarization rate is slow, thus requiring long lifetime storage vessels for the polarized gas. The typical time constant for polarizing large volume cells is 10 h to 20 h, hence relaxation times of 100 h or longer are desirable. Since the polarization rate is proportional to rubidium density, and substantial laser power is required to maintain high Rb polarization in the optically thick vapor, efficient optical pumping is essential for large volume spin filters. The laser power required can be decreased (or the polarization rate for a given laser power increased) by the use of alkali mixtures. We discuss each of these topics below.

### 2.1 Temperature-Dependent Relaxation

**Fig. 1:**
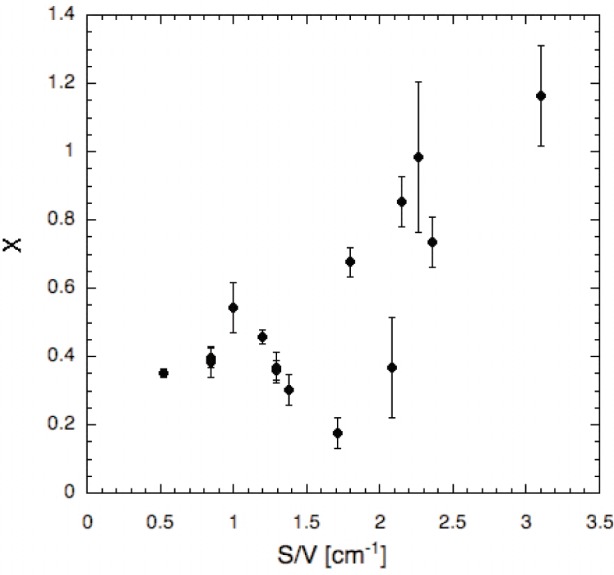
Measurements of *X* (see Eq. (1)) as a function of surface to volume ratio (S/V).

In an attempt to determine if the temperature dependence of the ^3^He relaxation is due to a larger than expected contribution from anisotropic spin-exchange or instead due to a temperature-dependent wall relaxation, we have determined *X* by directly measuring the dependence of the relaxation rate on Rb density. The results to date for cells of varying surface to volume ratio (*S/V*) are shown in Fig. 1. Although *X* varies between 0.2 and 1.2, no simple, clear dependence on *S/V* is apparent. While only cells with high *S/V* exhibit large values of *X*, some of these cells exhibit the lower values of *X* typical of cells with lower *S/V*. (However, it is currently not possible to say whether the lowest values of *X* are due to anisotropic spin-exchange.) These results indicate that anisotropic spin-exchange is not the only source of rubidium-density dependent relaxation, and that any surface related relaxation is not simply related to the geometric surface to volume ratio. One scenario is that there is a Rb density-dependent wall relaxivity that varies from cell to cell. For example, a relaxivity that varies by 30 % yields a 30 % variation in relaxation rate for *S/V* = 1 cm^−1^, but a 90 % variation for *S*/V = 3 cm^−1^. However, no correlation has been observed between room temperature relaxation rate and *X*, hence any such variation in the relaxivity that is relevant to hot cells appears to have a different cause than that which causes room temperature relaxation. Until the sources of this relaxation are understood and controlled, obtaining the highest polarization requires a cell to have not only a low room temperature relaxation rate *Γ*_r_, but also a low value of *X*. Fortuitously, the relatively large cells that will be needed for many neutron spin filter applications have moderate *S/V*, for which the lower values of *X* shown in Fig. 1 are typical. For experiments that require small cells, the yield for cell construction may be lower.

### 2.2 Cell development

Previously we reported the production of blown GE180 [19] glass spin-exchange cells^1^ with relaxation times (labelled *T*_1_) of several hundred hours [16, 20]. In some of these cells, wall relaxation was almost completely suppressed, resulting in relaxation times that approach the limit of 800/*P* h set by dipole-dipole interactions, where *P* is the room-temperature pressure of the cell in bar. [21] GE180 glass was chosen for neutron applications of SEOP because it has low permeability and does not contain boron. Low permeability is required to prevent loss of ^3^He gas for long term operation at the typical temperatures required for SEOP (170 °C.). To date the longest relaxation time that we have observed is 3000 h (determined with 500 h of data) for a 3 cm diameter GE180 sphere (identified as “Diamond”) filled with a partial pressure of 0.13 bar of ^3^He and a total pressure of 0.9 bar. (^4^He gas was added to avoid relaxation due to magnetic field gradients.) Although the ^3^He partial pressure is too low to be practical for a neutron spin filter, cells in the low pressure regime provide a direct test of the limits of achievable relaxation time.

Blown cells are suitable for experiments that do not require highly uniform polarization, such as the npdγ experiment. While the best relaxation times are obtained with such cells, experiments to measure beta-decay correlation coefficients require flat windowed cells to obtain a highly uniform path length. In previous publications, [16] we reported that the use of blown glass for the cylindrical cell body and/or the use of a nitric acid rinse [21] before filling the cells allowed us to increase typical relaxation times of flat-windowed cells from tens of hours up to ≈100 h. However, it does not appear that this approach will provide a reliable method to suppress wall relaxation sufficiently to eliminate its effects on the achievable polarization. Nevertheless, the relaxation times currently possible will not substantially limit the achievable polarization for practical spin filter cells. We return to this subject in our discussion of alkali mixture cells below.

Several effects related to the dependence of wall relaxation in SEOP cells on the sign, strength, and history of the magnetic field have been reported recently [22–24]. These effects are related to the presence of rubidium in the cell and in some cases also to the heating of cells that is required in the SEOP method. The origin of these unexpected effects is unknown and suggest some unknown form of magnetism that is either associated with impurities carried in during distillation of the Rb or from the Rb itself. There are practical consequences for spin filters. The *T*_1_ of SEOP cells depends on magnetic field strength, and the nature of the behavior depends on the type of glass used to make the cell [23]. SEOP cells exhibit “*T*_1_ hysteresis,” a significant dependence of wall relaxation at a fixed low applied field to previous exposure of a cell to a much larger field. [24] These phenomena are relevant to experiments in which a ^3^He spin filter may be used at or exposed to fields stronger than the typical value of ≈3 mT used for SEOP. A dependence of the relaxation time of a cell on its orientation in a low magnetic field has recently been reported, and changes in *T*_1_ following heating have been observed [22]. Both of these effects can produce changes in the expected performance and evaluation of a spin filter.

### 2.3 Spectrally Narrowed Diode Lasers

We have recently reported increased efficiency for spin-exchange optical pumping using spectrally narrowed diode array bars [26] instead of commercial fiber-coupled broadband diode lasers [16]. In that work we found that 14 W of spectrally narrowed laser light was sufficient to yield 70 % to 75 % ^3^He polarization in cells ≈100 cm^3^ in volume and it was confirmed that the rubidium polarization was close to 100 % in these cells. For larger cells (up to 640 cm^3^ in volume), the polarization was limited to 55 % to 60 % at this power level. We have recently implemented a 40 W diode array bar that allows us to deliver 25 W of spectrally narrowed laser light to a cell. Using an optical spectrum analyzer with an instrumental linewidth of 35 GHz, [25] we measured the linewidth of this laser to be 80 GHz (0.17 nm). Using this laser along with 25 W of broadband laser light entering the opposite side of the cell, we obtained the results listed in Table 1. The broadband laser was used because we found that 25 W of spectrally narrowed light was not sufficient to maximize the polarization in all cells. In the future we intend to use only spectrally narrowed laser light. Blown glass cells were pumped through their sides to avoid lensing from the non-uniform thickness of the blown ends, while the cell Spock was pumped through its ends. In essentially all the cells we reach 75 % ^3^He polarization and expect that the rubidium polarization is close to 100 %. In the largest cell (Astro), the ^3^He polarization is slightly reduced, which we believe indicates the onset of insufficient laser power to maintain 100 % rubidium polarization. In addition, in the flat-windowed cell tested that has a relatively short relaxation time (Spock), the ^3^He polarization is also slightly reduced. We have not necessarily optimized the use of the light for each cell. Several of the cells listed were constructed for the npdγ experiment, which requires polarization of a 10 cm diameter beam. The use of two spectrally narrowed diode array bars on each side of a cell should allow for 75 % ^3^He polarization in cells approaching one liter. However, the hybrid spin-exchange method discussed in the next section may be a better approach for very large cells and/or cells with relatively short lifetimes.

One drawback of spectrally narrowed lasers is that they are not as compact and convenient as commercial fiber-coupled lasers. Recently a new method for spectral narrowing of diode lasers has been reported [27], which may provide one step in convenience. In this approach a frequency selective element is attached to the diode laser array, hence no external optical elements are required. We expect to test such a system soon.

### 2.4 Hybrid Spin-Exchange Optical Pumping

For SEOP cells in which 1) the alkali polarization can be maintained near 100 %, 2) the spin-exchange rate is much slower than the room temperature relaxation rate, and 3) the value of *X* is in the empirically observed lowest range of 0.2 to 0.4, we have shown that 75 % ^3^He polarization is achievable. To obtain further improvements requires reducing the unexplained temperature dependence of the ^3^He relaxation. However, obtaining 100 % Rb polarization still requires substantial laser power for large cells, even with spectrally narrowed laser light. In addition, we have not yet established a method to obtain very long relaxation times in flat-windowed cells and in actual use relaxation times may be limited by magnetic field gradients [28]. (At a pressure of 1 bar, a field gradient of 1 × 10^−3^ cm^−1^ will yield a relaxation time of 60 h.) Hence a method to increase the spin-exchange rate is desirable, which leads to hybrid SEOP.

Although spin-exchange optical pumping has almost always been performed using rubidium, other alkalis have been proposed [29]. While the spin-exchange rate constants are very similar for potassium, sodium, rubidium and cesium [18], both the alkali-alkali and alkali-buffer gas spin-relaxation rate constants are larger in the higher-*Z* atoms due to increased spin-orbit interaction [13, 30]. (For this reason, cesium has never been expected to be practical for SEOP.) In the pressure range near one bar, alkali-alkali collisions dominate the alkali spin relaxation rate. The Na-Na spin relaxation rate has recently been reported to be 2 to 5 times lower than the K-K rate and 15 to 30 times lower than the Rb-Rb rate [31]. The lower spin-relaxation rates for K and Na would allow operation at higher alkali densities, thus providing shorter time constants for optical pumping, which would permit higher polarization in large cells in which the achievable polarization is limited by laser power. However, high power diode array lasers are not available for the wavelengths needed for direct optical pumping of either potassium (770 nm) or sodium (589 nm). Recently, a hybrid approach has been demonstrated [32], in which a potassium-rich mixture of rubidium and potassium is distilled into an SEOP cell, and the rubidium is optically pumped. Electronic polarization of the potassium atoms occurs on a rapid time scale due to Rb-K spin-exchange collisions, hence high potassium polarization can be maintained. Since the spin-relaxation rate of the potassium is much lower than that of rubidium, the same density of rubidium in a mixture cell can provide a much higher total alkali- ^3^He spin-exchange rate as compared to a cell with only rubidium. In such a cell, the potassium density should be much lower than the rubidium density, despite the much higher vapor pressure of rubidium. Since the vapor pressure of each alkali is weighted by its mole fraction, this can be accomplished by distilling a small amount of rubidium and a much larger amount of potassium into the cell. A substantial improvement in optical pumping rate was reported with a modest potassium to rubidium ratio of only 1.5 [32]. At a temperature of 244 °C, a 22.5 cm^3^ cell filled to a pressure of 3.6 bar was polarized to 73 % with a spin-up time constant of 2.5 h, twice as fast as that obtained in a pure Rb twin cell. The potassium polarization was close to 100 %, which suggests that the value of *X* is linked to total alkali density, not just rubidium density. Although unexplained limitations on the potassium polarization were observed at high K/Rb ratios, these effects were not found to be significant over a wide range of modest ratios. Given that the K/Rb ratio is not well controlled during current distillation procedures, this insensitivity to the ratio is a useful feature. Based on these promising results, we will be preparing Rb-K hybrid cells comparable in size to those listed in Table 1 to evaluate the potential improvement in pumping rate achievable.

Sodium may hold even greater promise for hybrid SEOP [32], but has technical challenges. First, essentially all glasses blacken over time in the presence of sodium at the higher temperatures required for sufficient sodium density (300 °C). Sapphire cells may avoid this problem. Although sapphire is birefringent, this issue can likely be addressed with optics to compensate for the birefringence. Since sapphire is the crystalline form of Al_2_O_3_, it would have very little neutron absorption or scattering. Perhaps the greatest challenge for neutron spin filters is the construction of large diameter cells from sapphire. A second issue for constructing Rb-Na hybrid cells is the substantial difference in vapor pressure. In the temperature range of interest, the Na/Rb vapor pressure ratio is 200, hence it is necessary for the alkali mixture to contain only ≈0.1 % Rb. Controlling the mixture ratio may prove to be difficult and require new methods. We are developing the diagnostics for measuring sodium density at the Univ. of Wisconsin, and the cell fabrication capability at NIST. To date, we have constructed three 7 cm diameter spherical cells, two from GE180 and one from Corning 1720 [33]. Studies with these cells will be forthcoming and we plan to fill a 3 cm diameter, 5 cm long flat-windowed sapphire cell soon.

For the first of the two GE180 cells, we obtained a mixture much too rich in Rb. However, this cell does yield a test of the effect of a sodium coating on wall relaxation and *X*. For optical pumping at 185 °C we measured a ^3^He polarization of 76 %, which implies that *X* = 0.29 (assuming 100 % Rb polarization), similar to results obtained with pure Rb cells. The relaxation was measured to be 650 h, close to the dipole-dipole limit for its fill pressure of 0.9 bar. Hence we conclude that sodium certainly has no detrimental effects on the relaxation time. For the other GE180 cell the relaxation time was 200 h, while the Corning 1720 cell had an atypically low value of 3 h. We have some preliminary indications that heating these cells to 300 °C to 350 °C may improve the relaxation time and that the presence of Na is required for this improvement to be observed. Hence we will also be studying the use of sodium in conjunction with heating as a cell preparation procedure. The blackening issue must still be addressed for this procedure to be practical. Note that if sodium is used simply as a coating, not for hybrid SEOP, the temperatures typically used for pure Rb cells would be used for optical pumping.

## 3. Conclusion

The development of large, long lifetime cells made from low permeability, boron-free glass along with the use of spectrally narrowed diode lasers for SEOP has made continuous operation of neutron spin filters with 75 % ^3^He polarization realistic. Further improvement will require reducing or eliminating the temperature dependent ^3^He relaxation that appears to be a ubiquitous phenomenon in SEOP cells. Suppressing wall relaxation in flat-windowed cells is a continuing challenge, but current performance is acceptable. The sign, magnitude and history of the magnetic field, as well as temperature cycling, can affect wall relaxation in SEOP cells. The practical issues for maximizing the ^3^He polarization in large cells and/or cells with reduced lifetime due to wall relaxation or stray magnetic fields can be addressed with hybrid SEOP.

## Figures and Tables

**Table 1:** Results for optical pumping of large diameter SEOP cells with 25 W of spectrally narrowed laser light incident on one side of the cell and 25 W of broadband laser light incident on the opposite side of the cell. All cells are blown GE180 glass cylinders, except for the cell Spock, which has polished GE180 windows optically sealed to a blown GE180 cylindrical body. The cell parameters listed are diameter *D* in cm, length *L* in cm, volume *V* in cm^3^, room temperature pressure *P* in bar, relaxation time *T*_1_ in h, and ^3^He polarization *P*_He_

Cell name	*D*	*L*	*V*	*P*	*T*_1_	*P*_He_
Nurse Chapel	7.2	6.5	260	1.25	330	0.75
Chekhov	8.1	6.0	310	1.27	340	0.77
Dino	10.6	5.1	452	0.9	530	0.74
Pebbles	11.2	5.2	508	0.87	350	0.75
Astro	11.3	6.4	640	0.89	735	0.72
Spock	10.3	7.4	620	0.90	70	0.64

## Footnotes

1 Certain commercial equipment, instruments, or materials are identified in this paper to foster understanding. Such identification does not imply recommendation or endorsement by the National Institute of Standards and Technology, nor does it imply that the materials or equipment identified are necessarily the best available for the purpose.
